# Editorial Notes

**Published:** 1946

**Authors:** 


### Editorial Note

**Sir Philip Robert Morris.**

The Bristol Medico-Chirurgical Society extends a warm welcome to the new Vice-Chancellor of the University, Sir Philip Robert Morris, C.B.E., M.A., who has been since 1944 Director-General of Army Education. He was born at Hildenborough, Kent, and educated at Tonbridge and Trinity College, Oxford. After teaching history and classics at Westminster Training College, he became Administrative Officer, Assistant Director, and eventually Director of Kent County Education Authority. He was awarded the C.B.E. in 1941, and was knighted in June, 1946. To a man of his energy and administrative ability it is probably a matter for congratulation rather than commiseration that he enters upon his responsible office in Bristol at a time when so much of the precious metal is fluid in the crucible, and ready to be poured out into fresh moulds, shaped according to the mind of the craftsmen. In no department of University Education is this more true than in the Faculty of Medicine. Probably no other Faculty will make such demands on his time and attention. The school has to be expanded to admit many more medical and dental students to meet the growing needs of the country. Fresh buildings and fresh staff are required. New professors and heads of departments have been, or will soon be, appointed. The medical curriculum is being modified. Some subjects, notably pædiatrics and psychiatry, are in need of development. Diplomas in public health, radiology and other subjects are under consideration. There are urgent problems of collaboration to be worked out between the voluntary hospitals, the public health authorities and the university. The university is now entrusted with the responsibility of giving advice to hospital governors all over the south-west of England when new members of staff are to be appointed. Postgraduate medical education is more necessary than ever now that so many doctors are being demobilized. These are only some of the problems that have to be tackled, and Sir Philip Morris has already shown an active and welcome interest in them. It is a great task that he has undertaken, but the possibilities for good are vast and far-reaching. He has our most sympathetic support.

